# Alginate and Chitosan-Based Delivery Systems for Improving the Bioavailability and Therapeutic Efficacy of Curcumin

**DOI:** 10.3390/pharmaceutics16030423

**Published:** 2024-03-19

**Authors:** Anand A. Sable, Amit Kunwar, Atanu Barik

**Affiliations:** 1 Radiation and Photochemistry Division, Bhabha Atomic Research Centre, Mumbai 400085, India; aasable@barc.gov.in; 2 Homi Bhabha National Institute, Anushaktinagar, Mumbai 400094, India

**Keywords:** curcumin, drug delivery system, nano-formulation, alginate, chitosan

## Abstract

One of the major challenges in harnessing the therapeutic benefits of curcumin (an active ingredient from turmeric) is its poor bioavailability due to its short biological half-life. In this regard, nanoformulations have shown tremendous hope for improving the pharmacokinetic and therapeutic behavior of curcumin by altering its biological stability and bioavailability. Biopolymers, especially alginate and chitosan, have received special attention as excipients to prepare nanoformulations of curcumin due to their abundant availability, biocompatibility, and amicability to form different types of self-assembled structures and ease of undergoing chemical modifications. However, there are certain challenges, such as poor water solubility under physiological conditions and heterogeneity with regard to molecular weight and large-scale production of well-preserved nanostructures. Substantial advancement has been achieved towards overcoming these challenges by developing newer derivatives through a chemical modifications approach, and this has ascertained the suitability of alginate and chitosan as excipients for drug delivery systems (DDS). The present minireview briefly discusses curcumin and its limitation as a drug molecule, carbohydrates as DDS, and the recent developments related to the alginate and chitosan-based nanoformulations of curcumin. Special emphasis has been given to highlighting the impact of alginate and chitosan-based nanoformulations in improving the therapeutic efficacy and bioavailability of curcumin.

## 1. Curcumin and Its Limitations as a Drug Molecule

**Figure 1:**
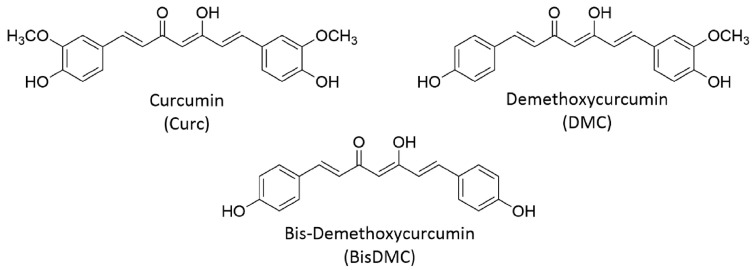
Major curcuminoids present in turmeric powder. Reproduced with permission from [3] (*Food Chem. Toxicol.* **2022**, *166*, 113254).

Turmeric powder is derived from the dried rhizome of a plant called *Curcuma longa*. The use of turmeric powder as a spice or food coloring agent dates back ~5000 years ago [1]. Apart from being a food additive, turmeric has also been in use since ancient times as a household medicinal remedy for the cure of a number of inflammatory illnesses in Southeast Asian countries. Around the middle of the 15th century, European explorers introduced turmeric to Europe and western countries [2]. Curcumin, a bright yellow compound along with two of its analogs, namely demethoxycurcumin and bis demethoxycurcumin, are the major active constituents of turmeric powder and are collectively called curcuminoids (Figure 1). Among the curcuminoids, curcumin is the most abundant fraction (~70%), followed by demethoxycurcumin (~17%) and bis demethoxycurcumin (~3%) [3,4,5,6].

**Figure 2:**
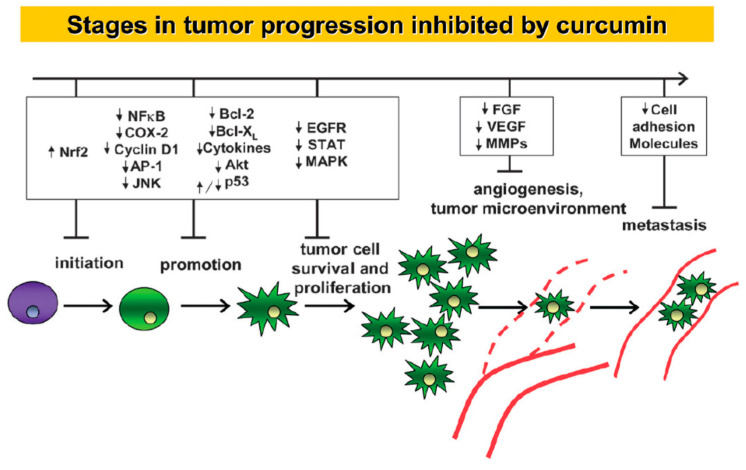
Stages of tumor progression inhibited by curcumin as the associated molecular targets are presented (↑—Upregulation, ↓—Downregulation). Reproduced with permission from [11] (*Cell. Mol. Life Sci.* **2008**, *65*, 1631–1652).

Curcumin, in its pure form, can be obtained by extraction from turmeric powder as well as by chemical synthesis [7]. Chemically, curcumin is a beta diketo-based compound and undergoes rapid keto–enol tautomerism when dissolved in solvents (Figure 1). Curcumin is practically insoluble in water at neutral pH. However, to some extent, it is soluble in alkaline media where the ionizable phenolic and enolic protons facilitate the dissolution. In common organic solvents such as methanol, dimethyl sulfoxide, acetonitrile, and acetone, it is fairly soluble. It is also soluble in lipids, proteins, micelles, and other organized assemblies wherein curcumin experiences a hydrophobic environment. Curcumin shows strong optical absorption and fluorescence in the visible region, which is useful for its detection by routine analytical and sophisticated instruments. The biological activities of curcumin are innumerable. For instance, it acts as an antioxidant, anti-inflammatory, anticancer, chemopreventive, antiparasitic, and antimalarial agent [8]. Similarly, a large number of biological targets have been identified that contribute to the biological activities of curcumin [9]. The most studied biological effect of curcumin in recent times is its ability to inhibit the growth of cancer cells at all three stages of carcinogenesis such as initiation, promotion, and proliferation [10]. The focused research in this direction using preclinical model systems has conclusively established that curcumin targets/modulates several signaling pathways, as shown in Figure 2, to exert the anticancer effect [11,12,13]. Most importantly, it is desired that any anticancer agent show selectivity towards cancer cells, and in this context, it is worth mentioning that curcumin is well known for its selective toxicity to cancer cells without affecting healthy cells [14]. Indeed, curcumin is highly safe for consumption, and even an oral dose of 12 g/day for healthy humans does not cause any adverse side effects [15]. The US FDA has recognized curcumin as a GRAS (generally recognized as safe) material. There are a number of curcumin-based nutraceuticals available on the market to improve or maintain the general well-being of humans. Despite showing excellent pharmacological activity against cancer, as well as many other disease models in preclinical studies, curcumin has not shown much success for said indications in clinical studies [16]. This has limited its translation from a “wonder molecule” to a “wonder drug”. The major reason for the limited clinical success of curcumin is the poor bioavailability due to low aqueous solubility and stability and faster metabolic degradation [17,18]. Therefore, the recent trend in the field of curcumin research is to design novel strategies to improve its bioavailability.

## 2. Drug Delivery System (DDS)

The major challenges faced by drug molecules in the complex biological environment are chemical instability, lack of cytospecificity, shorter circulation half-life, and rapid renal clearance [19]. Accordingly, the purpose of DDS is to encapsulate the drug of interest into a matrix to improve its chemical and biological stability, facilitate controlled release in biological environments, and impart selectivity toward the target site [20]. Recently, nanotechnology has captured the imagination of researchers to achieve this goal. Most of the DDS reported in the literature currently are nanocarriers [21]. The sizes of DDS in the nano-regime often have the added advantage of an enhanced permeability and retention (EPR) effect, particularly for chemotherapeutic drugs (a mechanism often referred to as passive targeting) [22]. Additionally, nanocarriers can also be functionalized with homing ligands to specifically recognize target cells (active targeting) [22]. The scope of DDS is not only restricted to the noncarriers, but it also encompasses many other systems such as gels, emulsions, macroparticles, micelles, etc. [23]. A variety of materials, such as inorganic metal-based nanosystems, lipid-based nanosystems, synthetic polymers, and a host of naturally occurring bio-polymers, including proteins, carbohydrates, and nucleic acids are reported in the literature for the fabrication of DDS [24]. Similarly, the synthetic methodologies of preparing DDS have also seen tremendous improvement with regard to achieving control over the size, shape, surface functionalization, and biological compatibility [25].

A large number of DDS have been explored in the recent past to improve the bioavailability and therapeutic effects of curcumin [26,27]. Most studies have shown encouraging results during the preclinical investigations, and a few of them have even progressed to the clinical trial stage to achieve higher bioavailability of curcumin. In this context, Hegde et al. have recently analyzed the outcomes of several clinical trials aimed at evaluating different curcumin formulations for better bio-availability [28]. Based on this analysis, authors have classified curcumin formulations (which showed improvement in bioavailability, absorption, and cellular uptake during clinical trials) under three major categories, viz., first, second, and third generation, depending on the type of excipients or carriers used in the formulations. Of these, the first-generation curcumin formulations have mainly employed turmeric fiber, edible oils, and piperine as the carriers. Such formulations have shown enhanced bioavailability compared with pure curcumin, mainly by inhibiting curcumin metabolism. The second-generation curcumin formulations have employed micelles, micro/nano-emulsions, nanoparticles, liposomes, and nanogels as carriers to increase the aqueous solubility and stability of curcumin and have shown enhanced bioavailability compared with pure curcumin through increasing membrane permeability and cellular uptake. The third-generation curcumin formulations are mainly based on the nutraceutical approach wherein curcumin is noncovalently mixed with food-grade excipients to improve its bioavailability as well as to show synergism towards the health benefits. Such formulations have also shown enhanced bioavailability compared with pure curcumin through increased membrane permeability and cellular uptake. Nevertheless, scientific communities still remain skeptical about the therapeutic utility of not only curcumin per se but also of encapsulated curcumin in human clinical trials [29].

## 3. Carbohydrate as Excipient for DDS

Among polymeric materials, natural polymers, in general, have the advantages of superior biocompatibility and low toxicity compared with synthetic polymers, but with synthetic polymers, one can have better control over the precise molecular weight, suitable chemical modification, tunable mechanical properties, and reproducibility [30]. Carbohydrates are one such class of natural polymer that has been extensively investigated as DDS. Carbohydrates have an empirical formula, C_n_H_2n_O_n_, where every carbon atom is associated with one molecule of water [31]. The word ‘carbohydrate’ is a combination of carbo (meaning carbon) and hydrate (meaning water), i.e., hydrated carbon. Monosaccharides are also considered the basic units (monomers) from which all other carbohydrates, such as disaccharides (two monosaccharide units), oligosaccharides (three to six monosaccharide units), and polysaccharides (more than six monosaccharide units) are built through glycosidic bonds. Polysaccharides are the most abundant biopolymers in nature, which account for more than 80% of all biopolymers. They are mostly used as energy storage and structural materials [32]. Further, they are involved in cell recognition, differentiation, adhesion, and other important biological processes. Along with other natural biopolymers such as lipids, proteins, and nucleic acids, polysaccharides provide unmatched prospects for targeted delivery of drugs. Some of the unique features of carbohydrates in general are (i) abundant availability, (ii) chemical diversity, (iii) biocompatible/biodegradable nature, (iv) natural targeting ability, (v) protein-repellent, (v) extremely water soluble, and (vi) reduced tendency for agglomeration [33]. The chemical diversity of carbohydrates arises during the formation of O-glycosidic bonds through chain elongation and branching, which is not limited to a fixed position as observed for proteins and nucleic acids [34]. For instance, a combination of twenty amino acids yields 6.4 × 10^7^ hexapeptide isomers, whereas an oligosaccharide with the same number of hexose units yields 1.44 × 10^15^ isomers. Thus, each of the isomers can act as a scaffold for DDS. Moreover, the recent literature indicates that polysaccharides can be conjugated with proteins to produce unique biomolecular structures (PRO-POL), which in turn can be used to fabricate DDS with improved bio-compatibility, drug encapsulation and drug release kinetics [35]. The proteins and carbohydrates are combined either through the covalent conjugation or through the noncovalent interactions. The hydrophobic protein moiety in such conjugates acts as a binding moiety for hydrophobic drugs such as curcumin, whereas the carbohydrate moiety helps in the interaction of DDS with the biological tissues. Due to the vastness of the scope of work, the present review article aims to restrict the discussion on a recent development related to the alginate and chitosan-based DDS for curcumin and the important progress achieved so far from the preclinical studies.

## 4. Alginate Based DDS for Curcumin

**Figure 3:**
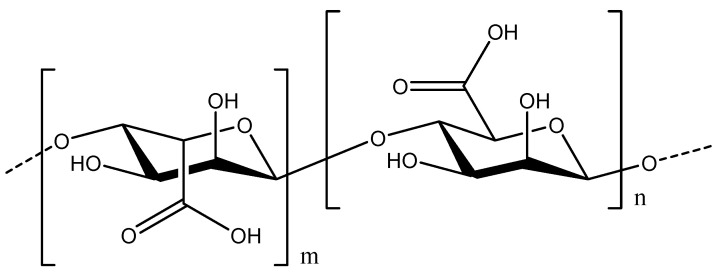
Chemical structure of alginate.

Alginic acid is a natural polysaccharide found in the cell walls of brown algae [36]. It is arranged in a linear fashion comprising an anionic block co-copolymer of β-1,4-linked mannuronic acid (M block) and *α*-1,4-linked guluronic acid (G block) (Figure 3). Depending on the algae species, the arrangement of the M or G blocks may be the same or alternating. The physicochemical properties of alginates are very much dependent on M/G ratios, length of blocks, and arrangement of repeating units in the biopolymer [37]. Alginate is one of the most commonly used polymer matrices for preparing nanoformulations due to its nontoxic, biocompatible, and biodegradable nature and easy gelation ability [38]. The delivery of curcumin through alginate has been reported mainly in the form of hydrogels, conjugates, self-assembled micelles, edible film, emulsion, microparticles, and nanoparticles [39,40,41].

Alginate-based hydrogels and sponges are easily prepared by the ionic gelation method using divalent cations [42,43,44,45]. These alginate gels and sponges are bio-adhesive and readily attach to tissue surfaces when sufficiently hydrated. This property of alginate hydrogel/sponge is useful in preparing wound dressing and bioprinting materials. It has been observed that a high content of G blocks in the polymer backbone forms rigid hydrogels due to egg-box conformational arrangement. However, a high content of M blocks in the polymer backbone results in the formation of soft gels, which are less adhesive in nature. In a recent development, it has been observed that freeze dying followed by compression significantly enhances the mechanical strength of alginate sponges [44]. Interestingly, such lyophilized compressed sponges have shown excellent efficiency as a hemostat and also as an anti-peritoneal adhesion barrier in laboratory studies. These properties may find potential applications for preventing post-operative adhesion and hemostasis in clinics. The bilayer alginate sponges show higher hemostatic and antiadhesion properties compared with a single layer, suggesting that the bilayer structure is required for these properties [44]. Moreover, the hemostatic and antiadhesion properties of the bilayer alginate sponges are primarily governed by their thickness, and ~100–200 µm is reported as the optimum thickness to show hemostasis and anti-peritoneal adhesion simultaneously [44]. Together, the adhesive property of alginate hydrogel/sponge can be tuned as per the requirement for a particular biomedical application. Further, alginate can also be crosslinked with protein, lipid, drug molecules, or certain functional groups in order to tune the mechanical strength and promote the self-assembly of hydrogel [46]. The incorporation of graphene oxide in alginate hydrogels has received special attention with respect to its stability. It has been shown that the presence of graphene oxide exerts excellent biocompatibility and stability to the hydrogel matrix in aqueous media by preventing the de-crosslinking mechanism of the hydrogel network [47,48]. Additionally, the presence of graphene oxide in the alginate hydrogels also improves the release kinetics of entrapped drugs (such as curcumin) by favoring slow and sustained release [47,48].

The loading of curcumin within alginate hydrogels is performed either directly as a drug molecule or as a nanoformulation [49]. The encapsulation of curcumin within hydrogels in a nanoformulation provides better control in terms of release kinetics and pharmacokinetics. There are quite a few reports where curcumin nano-emulsions have been encapsulated through an ionic gelation technique using an alginate matrix and calcium ions [50,51]. In these studies, a self-emulsifying curcumin solution prepared by mixing curcumin with surfactant and emulsifying agent was loaded into the alginate beads of size ~1.5 mm [50]. The encapsulation efficiency was dependent on several factors, such as the amount of sodium alginate, calcium ion concentration, and even the physical nature of curcumin before encapsulation. It was observed that encapsulation efficacy was higher when self-emulsifying curcumin was used compared with curcumin powder. The curcumin-loaded alginate beads were proposed for oral delivery [50]. As discussed earlier, the metabolic degradation of curcumin in the gastrointestinal tract is a major concern for its poor bioavailability. Therefore, the major aim of oral-based DDS for curcumin is to improve its intestinal absorption in a stable form. Accordingly, understanding the release of curcumin from alginate beads under simulated biological fluids conditions is also an important parameter for drug delivery applications. Notably, the studies indicated that more than 80% of curcumin was released within eight hours in simulated colonic fluid (SCF) having pH > 7.4, whereas only a small amount of curcumin was released in the simulated intestinal fluid (SIF) and simulated gastric fluid (SGF) having pH 6.8 and 1.2 respectively. This clearly confirmed the stability of alginate beads in the acidic environment of the stomach and the preferential degradation followed by the release of curcumin under the alkaline environment of the intestine, which is desirable for the maximum absorption of curcumin in the stable form [50]. Recently, Ding et al. employed a similar strategy to fabricate curcumin-loaded zein-alginate nanogels with a core-shell-like structure [52]. Briefly, curcumin-loaded zein nanoparticles (Cur@ZA) were prepared using the anti-solvent precipitation method. Subsequently, it was coated with alginate through calcium ion-induced crosslinking. The size of the assembly was dependent on the weight ratio of zein versus alginate. The optimized formulation prepared by using zein and alginate in the weight ratio of 2:1 exhibited a hydrodynamic size of ~472 nm and entrapment efficiency of >92% for curcumin with a zeta potential value of −16.0 ± 2.0 mV.

The formulation showed enhanced stability against aggregation in the aqueous medium. The superior stability of zein-alginate nanogel as compared with simple zein nanoparticles was attributed to a hydrophilic coating of alginate. Additionally, the comparative drug release studies in the simulated digestion conditions suggested that calcium ion complexed zein-alginate nanogel facilitated the slow and steady release of curcumin as compared with those fabricated without crosslinking. Thus, the study emphasized the potential of alginate-based coating for improving the stability and drug-release behaviors of composite nanostructures. In another study, a nano-emulsion of curcumin was obtained from spontaneous emulsification of curcumin, surfactant oil, and water with a size of ~25 nm. The curcumin nano-emulsion was subsequently packed in the spherical alginate hydrogel beads with a diameter of ~0.5 mm. These hydrogel beads showed a high encapsulation efficiency of 99.15% and a loading capacity of 7.25 mg/g for curcumin. The release of curcumin was pH-dependent, with higher release in the alkaline medium as desired [53]. In another study, Paswan et al. reported the fabrication of curcumin-loaded alginate-polylactic acid (PLA) hydrogel beads through an ionic gelation technique [54]. Briefly, curcumin was solubilized using the PLA, and the resulting solution was used to induce ionic gelation of the alginate matrix. The alginate-PLA beads showed excellent hemocompatibility, curcumin encapsulation efficiency of 81.47%, and higher release of curcumin under SCF (pH = 7.4) than in SGF (pH = 1.2) conditions. In all the above studies, the release of curcumin from alginate hydrogel at alkaline pH was attributed to the swelling of hydrogel followed by erosion. Although such studies have put forward a strategy of the preferential release of curcumin in the alkaline environment of the intestine in order to improve its bioavailability, the translation of such strategy under actual in vivo conditions can be argued considering that curcumin per se is unstable under alkaline environment. For instance, Kumavat et al. have systematically investigated the stability of curcumin in the aqueous solution of varying pH ranging from 1 to 7.4 and have clearly shown that curcumin degradation into ferulic acid and vanillin increases as the pH of the solution increases towards 7.4 [55]. Therefore, for the actual increase in bioavailability, the absorption of curcumin formulation from the intestine should be faster so that the residence time of curcumin in a physiological medium is minimal. In this context, nanoformulations have received greater attention. Indeed, alginate-based nanocarriers have also been evaluated to improve the bioavailability of curcumin in humans. For instance, Govindaraju et al. recently reported the enhanced water dispersibility and bioavailability of curcumin encapsulated in the alginate-polysorbate 80 nanoparticles [56]. The curcumin-loaded nanocarriers were synthesized using the ionotropic gelation method. The physicochemical properties of curcumin-loaded alginate-polysorbate 80 nanoparticles comprised particle size of 383 nm, zeta potential of +200 mV, and entrapment efficiency of ~95%. The formulation showed the release of curcumin under the influence of SCF (pH 7.4) but not under SGF and SIF. The human studies indicated that the formulation achieved five times higher bioavailability of curcumin than the plain curcumin suspension with maximum plasma concentration (C_max_) of 636 ± 122 ng/mL and 17.9 ng/mL for nano-formulation and curcumin suspension, respectively.

**Figure 4:**
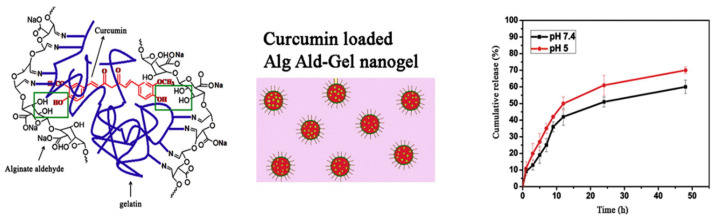
Representation of the possible interaction mechanism between curcumin and Alg Ald-Gel nanogel, postulated model of nanogel and in vitro curcumin release from Alg Ald-Gel nanogel at pH 5 and pH 7.4. The green box highlights the sites of intermolecular hydrogen bonding between the phenolic OH group in curcumin and free OH goup of Alg Ald-Gel nanogel. Reproduced with permission from [57] (*Mater. Sci. Eng. C* **2016**, *68*, 251–257).

Though the release of curcumin from an alginate matrix at alkaline pH is favorable for oral-based DDS, the same may not be an ideal rationale for designing an intravenous DDS for the chemotherapeutic application of curcumin. Instead, higher release at acidic pH compared with neutral pH is preferred for the selective delivery of curcumin in tumor cells owing to their acidic environment. The release behavior of curcumin from the alginate matrix can be tuned to an acidic environment by suitable modification of the alginate core. Sarika et al. recently reported a novel approach to achieve this objective (Figure 4). The authors first prepared alginate aldehyde (Alg-Ald) by performing the partial oxidation of alginate [57]. Subsequently, Alg-Ald was mixed with gelatin (Gel) to prepare nanogels of spherical morphology through an inverse mini-emulsion technique using span 20 as an emulsifying agent. The structural analysis of this assembly revealed crosslinking between Alg-Ald and gelatin, where the free amine groups in gelatin were covalently attached to aldehyde groups on oxidized alginates through Schiff’s base interaction. The synthesized Alg Ald-Gel nanogel was used for curcumin loading through the physical adsorption method. The typical size of curcumin-loaded Alg Ald-Gel nanogels was ~431 ± 8 nm with an encapsulation efficiency of ~72 ± 2%. Curcumin-loaded Alg Ald-Gel nanogels show a zeta potential of −36 ± 5 mV. The encapsulation of curcumin resulted from the strong intermolecular hydrogen bonding between the phenolic OH group in curcumin and unreacted OH functionalities on alginate aldehyde.

**Figure 5:**
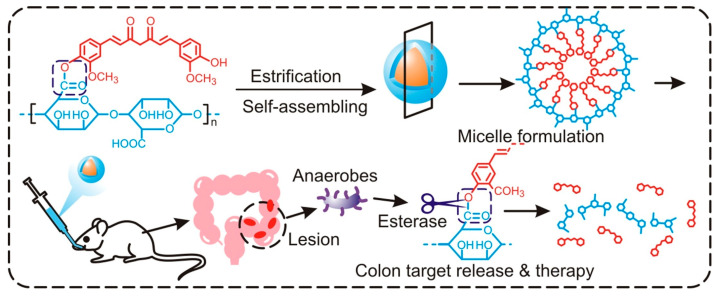
The scheme shows commensal flora triggered targeted release of curcumin from alginate-curcumin micelle for ulcerative colitis treatment. Reproduced with permission from [58] (*Colloids Surf. B:* **2021**, *203*, 111756).

Interestingly, the formulation showed a higher release of curcumin at acidic pH as compared with neutral pH due to the cleavage of the imine bond (Schiff’s base) between gelatin and alginate aldehyde. In agreement with the release studies, the curcumin-loaded Alg Ald-Gel nanogels exhibited significantly higher uptake and toxicity in breast cancer cells (MCF7). Drug conjugation directly with DDS has also gained prominence in recent times. On similar lines, the covalently conjugated curcumin with alginate (Alg-Cur) has been reported for the treatment of ulcerative colitis (UC), an inflammatory bowel disease (Figure 5). Briefly, curcumin was conjugated to alginate through esterification between the phenolic OH group of curcumin and the carboxylic (-COOH) group of alginates [58]. Due to the amphiphilic and hydrophobic nature of alginate and curcumin, respectively, the Alg-Cur conjugate self-assembled into a stable micellar nanocarrier in a biological environment with a zeta potential of −21.25 mV. After oral dosing, 92.32% of curcumin alginate conjugate reached the colon tissue in a mouse model. The release of curcumin from the conjugate was specific to the colon region where abundant esterase produced by commensal anaerobic flora cleaved the ester bond between curcumin and alginate, thereby releasing curcumin, as shown in Figure 5. The release of curcumin was correlated with its higher bioavailability and effective suppression of the inflammatory response. The formulation specifically inhibited TLR4 expression in colonic epithelial cells, reduced the transcription and expression of the pro-inflammation cytokines, and prevented the infiltration of lymphocytes, macrophages, and neutrophils.

**Figure 6:**
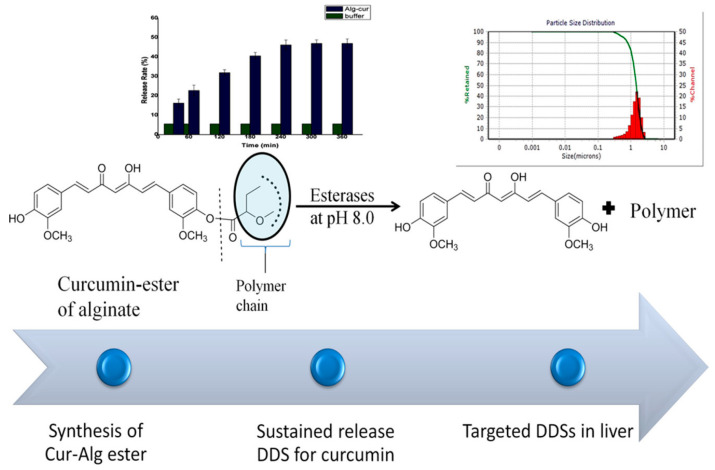
The figure shows the synthetic scheme of Cur-Alg ester, its particle size distribution, and the cleavage and release of curcumin by liver homogenate at pH 8. Reproduced with permission from [59] (*J. Mol. Struct.* **2023**, *1283*, 135307).

Another similar study [59] has shown the utility of curcumin conjugated with alginate through the modified ester linkage for specific delivery to liver tissues (Figure 6). The curcumin-alginate ester (Cur-Alg ester) was synthesized with ∼57% conjugation efficiency. The conjugate was characterized for crystal size and particle size distribution, and both parameters were found to be within an acceptable range for a nanocarrier to be an effective DDS. Further, the conjugate was stable during the in vitro simulated gastric and intestinal fluids. In contrast, the conjugate underwent cleavage to release curcumin in the presence of liver homogenate at pH 8. The specific cleavage of the conjugate by the liver enzymes prompted authors to project the conjugate as the liver-targeted prodrug. This study also highlights that a similar strategy can be employed to develop liver-specific DDS.

**Figure 7:**
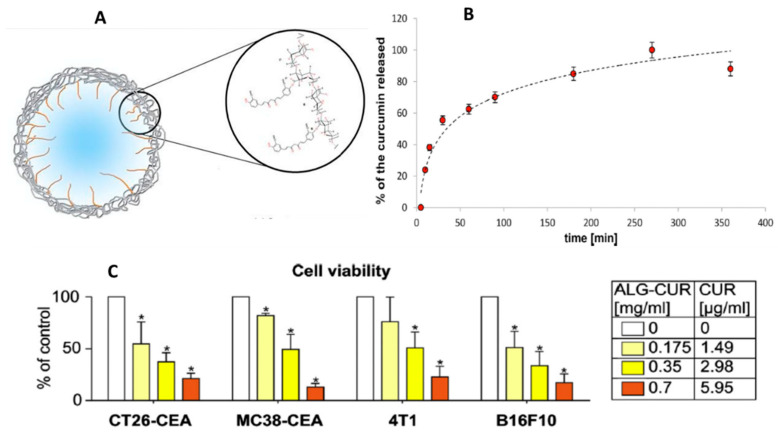
(**A**) Schematic illustration of the structure of bioconjugate AA-CUR and the micelle formed by conjugate AA-CUR (**B**) Curcumin release profile obtained for AA-CUR/oleic acid mixture at 37 °C, pH = 7.4 (**C**) Analysis of cytotoxicity of AA-CUR bioconjugate performed using different mouse cancer cell lines by MTT assay. * Statistical significance as compared to control (0 µg/mL of curcumin). Reproduced with permission from [60] (*Eur. Polym. J.* **2019**, *113*, 208–219).

In another study [60,61], covalently linked curcumin and alginate (AA-CUR) was demonstrated to mediate excellent anticancer activity (Figure 7). Briefly, AA-CUR conjugate was crosslinked with calcium and dispersed in an aqueous medium to favor the self-assembly of spherical micelles with a size of 100–200 nm with high colloidal stability (zeta potential −53 mV) above the critical micelle concentration (0.6 mg/mL). Curcumin was released from the conjugated system upon hydrolysis of the ester bond between the curcumin and alginate chain, and complete release was observed within 5 h under physiological conditions. Interestingly, the conjugate showed suitability for systemic administration as it was found to be non-toxic to red blood cells as well as peripheral blood mononuclear cells and mouse primary brain endothelial cells. On the other hand, the conjugate exhibited specific toxicities in the mouse cancer cell lines (CT26-CEA, 4T1, B16F10, and MC38-CEA) up to 80%. Together, the above studies established the potential of alginate-based bioconjugates of curcumin as an effective DDS to achieve desired therapeutic applications.

## 5. Chitosan Based DDS for Curcumin

**Figure 8:**
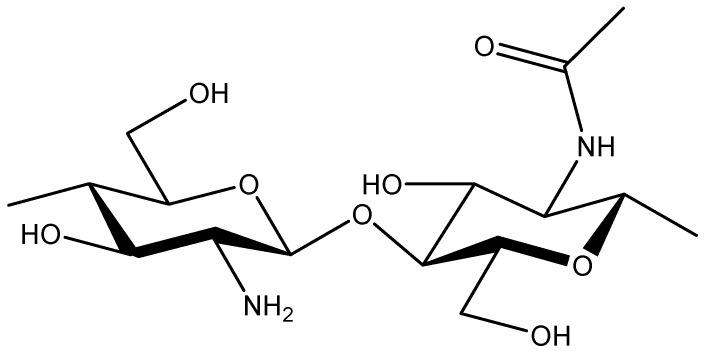
Chemical structure of chitosan.

Chitosan is the deacetylated product of chitin, a polysaccharide extracted from the shells of crabs, shrimps, shellfish, and lobsters [62]. Chitin is composed of a linear polymeric chain of 2-acetaylamino-2-deoxy-β-D-glucopyranose units connected through β-1,4 linkages (Figure 8). It is mostly insoluble in water at physiological pH but soluble in an acidic medium (pH < 6.5) due to the presence of the amino group. After solubilization in an acidic medium, chitosan becomes a hydrophilic cationic polymer that is able to form polymeric networks such as films or gels [62]. The availability of specific functional groups such as -NH_2_ and -OH groups in the chitosan renders it suitable for chemical modification, which expands the application of chitosan by improving its physico-chemical properties [63]. Some of the well-characterized chemically modified derivatives of chitosan are thiolated chitosan, acylated chitosan, N-trimethyl chitosan, and carboxymethylated chitosan [64]. Additionally, the physiochemical properties of chitosan can also be tuned by varying the molecular weight and degree of deacetylation. Given the cationic nature of chitosan, it has very low immunogenicity and excellent muco-adhesive ability. These properties favor the suitability of chitosan as a DDS for enhanced absorption or uptake of desired drugs into cells [65]. Self-assembly and chemical cross-linking are the most widely employed methodologies for the preparation of chitosan-based nanoformulations [66,67]. The self-assembly of chitosan is achieved by modulating its amphiphilicity. Chitosan, as such, is hydrophilic; however, through chemical modification such as acetylation, its hydrophobicity is increased, which in turn favors the self-assembly with hydrophobic moiety residing at the core and hydrophilic functional groups facing outside towards the water molecules [68]. The stability of the amphiphilic chitosan-based nanostructures is governed by hydrophobic forces. Similarly, other derivates of chitosan, such as methylated and carboxymethylated chitosan, have gained importance as excipients as they are soluble at neutral pH and thus provide an opportunity for the preparation of nanoformulations maintaining physiological conditions [69]. Apart from controlling amphiphilicity, polyelectrolyte complexation is also a strategy to induce self-assembly of chitosan [70]. Briefly, chitosan is a positively charged polymer at acidic pH, and therefore, it undergoes electrostatic interaction mediated co-assembly with other negatively charged polymeric materials such as proteins, carbohydrates, and synthetic polymers to form nanoparticles. Covalent crosslinking is another important methodology for preparing chitosan nanoformulations [71]. Glutaraldehyde, tripolyphosphate (TPP), dicarboxylic acid, and epichlorohydrin are the most widely used cross-linking agents for chitosan [72]. Of these, polyanion tripolyphosphate-mediated crosslinking or ionic gelation are preferred as the nanoformulations prepared using these methods do not show any intrinsic toxicity and retain the pharmacological activity of the entrapped drug [73]. Some recent studies on the preparation of chitosan nanoformulations as DDS for curcumin are discussed in the following section.

**Figure 9:**
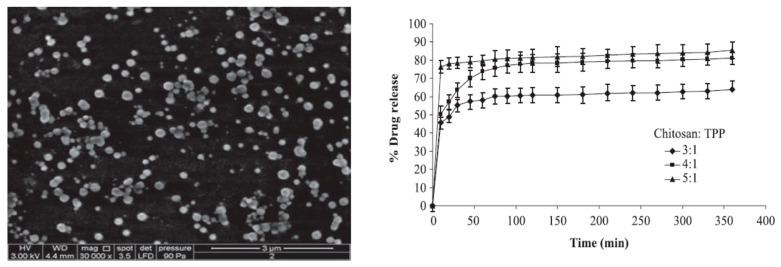
The images show FE-SEM image of nanoparticles prepared with chitosan: TPP ratio of 4:1 and drug release profiles of curcumin-loaded CS-NP as affected by cross-linking densities. Reproduced with permission from [74] (*Pharm. Dev. Technol.* **2013**, 18, 591–599).

Chuah et al. reported [74] the synthesis of chitosan nanoparticles with a size ranging from 261 to 412 nm by varying the ratio of chitosan and the polyanion tripolyphosphate as a crosslinking agent (Figure 9). It was observed that by tuning the pH of the solution, one could attain an optimum size of nanoparticles with high colloidal stability in terms of zeta potential (+38.1 mV). Curcumin-loaded chitosan nanoparticles displayed excellent mucoadhesive properties where it was suggested that apart from the -NH_3_^+^ group of chitosan, the phenolic OH group, and diketo group in curcumin also contributed to mucoadhesion through hydrogen bond formation. Further, it was observed that the loading of curcumin in the chitosan matrix remained more or less unaffected, but the release of curcumin from the chitosan matrix was very much dependent on the amount of crosslinking agent, i.e., tripolyphosphate. With a higher amount of tripolyphosphate, the chitosan nanoparticle assembly was more tortuous, and therefore, the release of curcumin from the nanoparticle assembly was also hindered.

In another study, a similar ion gelation methodology was employed to encapsulate curcumin as well as curcuminoids (a combination of curcumin, demethoxycurcumin, and bisdemethoxycurcumin) in the chitosan matrix. These nanoformulations had encapsulation efficiencies of more than 70% with sustained release of curcumin. Further, the formulations exhibited potential anticancer activity in human oral cancer cell lines and strong antibacterial activity against model systems [75,76].

Curcumin-loaded chitosan nanoparticles have also been reported as a novel therapeutic to combat arsenic-related toxicity [77]. In this formulation, nanoparticles of less than ~50 nm in size were prepared by crosslinking chitosan with glutaraldehyde. Curcumin is known for its excellent metal chelation properties [78,79]. Notably, the anti-oxidant and metal chelation properties of curcumin were retained after encapsulation into chitosan. In addition, the encapsulated form was more effective than the free crystalline curcumin. Further, the efficacy of curcumin-loaded chitosan nanoparticles against arsenic-induced toxicity was evaluated in a rat model. The results indicated that oral administration of curcumin-loaded chitosan nanoparticles at a dosage of 1.5 mg/kg body weight was able to significantly reduce the arsenic (2 mg/kg body weight)-induced oxidative stress (ROS, oxidized glutathione, thiobarbituric acid-reactive substance levels) in the brain tissue [77]. The formulation also augmented the level of anti-oxidant enzymes such as superoxide dismutase and catalase in the brain tissues and restored the brain functions in terms of the levels of dopamine, norepinephrine, and 5-hydroxytryptamine that were changed during exposure to arsenic.

**Figure 10:**
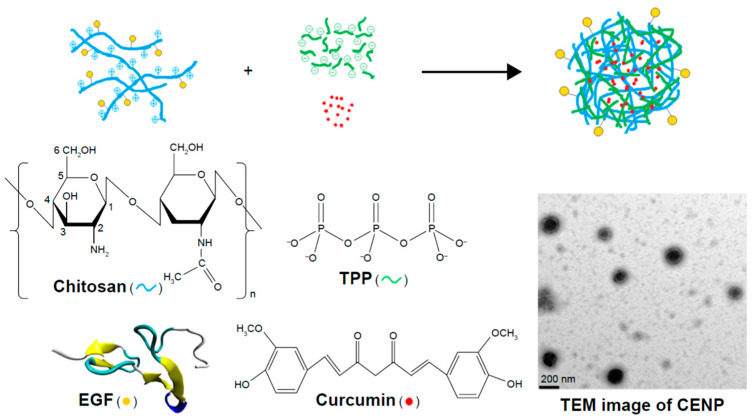
Schematic diagram for the nanoparticle assembly of CENP from EGF-conjugated chitosan, TPP, and curcumin. ENP—curcumin-encapsulated and EGF—conjugated chitosan/TPP nanoparticles; EGF—epidermal growth factor; TEM—transmission electron microscopy; TPP—tripolyphosphate. Reproduced with permission from [80] (*Int. J. Nanomedicine* **2018**, *13*, 903–916).

There are also interesting reports [80] related to the active targeting of curcumin through chitosan nanoparticles. For instance, curcumin encapsulated in epidermal growth factor (EGF) conjugated chitosan nanoparticle (CENP) was shown to exhibit promising anticancer effects against EGF receptor overexpressing cancer cells (Figure 10).

Briefly, EGF-conjugated chitosan was subjected to ionic gelation using polyanions of tripolyphosphate to prepare CENP nanoparticles. The prepared nanoparticles exhibited a hydrodynamic size of ~230 nm, a TEM size of ~160 nm, and a positive zeta potential value. Further, curcumin-loaded CENP exhibited significantly higher therapeutic efficacy (IC_50_ = 3.4 µM) compared with those encapsulated in simple chitosan nanoparticles (IC_50_ = 11.9 µM) in MKN45 cells in the presence of visible light. The nanoformulations in the above concentration range did not exhibit any dark toxicity, indicating their biocompatibility. The phototoxicity caused by curcumin-loaded CENP was attributed to the generation of singlet oxygen from the photoexcited triplet state of curcumin through a Type II mechanism [80]. Together, the above study provided the proof of concept that curcumin-loaded CENP might be an excellent system for photodynamic therapy.

Chitosan nanoparticles have also received attention for improving the bioavailability of curcumin. In this regard, Arozal et al. have reported the improved pharmacokinetic properties of curcumin-loaded chitosan-tripolyphosphate nanoparticles in a rat model [81]. The group prepared curcumin-loaded chitosan-NaTPP nanoparticles using ionic gelation methods, as described in the previous section. The particle size of the curcumin-loaded nanoparticles was ~11 nm. The entrapment and loading efficiency of curcumin in the nanoparticles were ~99.97% and ~11.34%, respectively. The curcumin-loaded nanoparticle showed superior mucoadhesive properties and an increase in area under the curve (AUC), maximum plasma concentration (C_max_), and half-life (t_1/2_ elimination) compared with free curcumin. Similarly, Sampathi et al. have recently reported the encapsulation of curcumin nanosuspension (Cur-NS) into chitosan-pectin microbeads as a novel strategy to enhance its oral bioavailability [82]. Briefly, Cur-NS synthesized by the anti-solvent precipitation method was entrapped within the chitosan microbeads through the ionic gelation method by using zinc chloride as a cross-linker and pectin as a rate-limiting polymer. The microbeads (Cur-NS-MB) were subsequently coated using hydroxypropyl methylcellulose. The coating was performed to protect the formulation from acid digestion in the stomach and intestine. The optimized formulations exhibited an average particle size of 193.5 ± 4.31 nm with a polydispersity index of 0.261 ± 0.020, entrapment efficiency of 99.45 ± 3.11%, loading efficiency of 14.54 ± 1.02%, and colon-specific curcumin release without undergoing gastric degradation. The animal studies indeed confirmed a 2.5-fold increase in C_max_ and a 4.4-fold increase in AUC^48h^ of curcumin delivered through formulation compared with free curcumin. Thus, Cur-NS-MB demonstrated the potential as a colon-specific DDS. In another study, Vijayakurup et al. evaluated curcumin-loaded chitosan nanoparticles for cellular uptake, pharmacokinetics, and in vivo efficacy as a chemopreventive agent against B[a]P-induced lung adenocarcinomas [83]. The curcumin-loaded chitosan-tripolyphosphate nanoparticles in a size range of 170–200 nm were prepared by the ionic gelation method. The in vitro studies indicated that chitosan nanocurcumin facilitated the sustained release of curcumin up to 180 h and also showed significantly higher cellular uptake in lung cancer cells. The in vivo studies employing a murine model confirmed improvement in the bioavailability of chitosan nanocurcumin in comparison with free curcumin in the lung tissue. Most remarkably, the chitosan nanocurcumin could prevent the development of B[a]P-induced lung adenocarcinomas even at a dose equal to one-fourth that of free curcumin. Further, authors have also reported the pharmacologic safety of the formulation through detailed chronic toxicity analysis. This study clearly established the supremacy of the formulation over free curcumin as a potential chemopreventive and oral supplement against environmental carcinogenesis.

In additional research reports, there are also a few patent publications on using chitosan-based nanoparticles as a strategy to improve the bioavailability of curcumin. For instance, a recent patent publication [84] deals with a method of preparing curcumin bound to chitosan nanoparticles and its evaluation to enhance curcumin bioavailability. Briefly, chitosan nanoparticles with an average size of ~60 nm were prepared by a spray drying method. The curcumin was physically adsorbed on the surface of chitosan nanoparticles by incubating the aqueous solution of chitosan nanoparticles with curcumin dissolved in alcohol. The unbound curcumin was removed from the reaction mixture by centrifugation. The bioavailability of curcumin-bound chitosan nanoparticles was evaluated in a mouse model. As per the claims, the oral administration of the invented formulation showed almost 10-fold higher bioavailability in circulation compared with curcumin administered through olive oil. The mice administered with curcumin-loaded chitosan nanoparticles also showed remarkably longer circulation half-life and, in turn, cured the mice of *Plasmodium yoelii* infection.

## 6. Conclusions and Future Directions

DDS is the need of the hour to utilize the medicinal values of curcumin. Both alginate and chitosan are biocompatible natural polymers with abundant availability. Due to the opposite ionic charge character, they readily form nano assemblies by polyelectrolyte complexation, which are stable and exhibit a stimulated release profile of curcumin and other bioactive compounds. The pH-dependent swelling behavior of alginate has been exploited for the release of curcumin in a neutral environment of the small intestine compared with the acidic environment of the stomach. However, to translate these effects into clinical trials to improve the therapeutic benefits of curcumin, there is still a need to optimize the conditions related to the faster and specific uptake of curcumin formulations within cells. In this regard, growing evidence from the preclinical studies indicates that alginate and chitosan-based nanoformulations have the potential to improve the cellular uptake, bio-availability, and therapeutic efficacy of curcumin. Accordingly, several curcumin-based nutraceutical and functional foods are already being developed using alginate and chitosan as excipients, and this is also evident from the growing number of intellectual property rights on this topic. However, there is barely any clinical evidence on alginate and chitosan-based curcumin formulations for improving bioavailability and/or therapeutic effects. One of the possible reasons for this could be the challenges associated with the scaling up of nano-formulations with controlled physicochemical properties. Thus, along with designing clinical-grade nano-formulations, there is a need to pay attention to the properties of carbohydrate polymers used as excipients and reaction conditions such as the amount of polymer, stirring rate, reaction time, temperature, pH, etc. Without optimization of all these conditions, the clinical translation of alginate and chitosan as DDS for curcumin with desired particle size, encapsulation efficiency, and loading efficiency may not be realized. Simultaneously, there is a need for extensive clinical studies of the newly designed alginate and chitosan-based DDS of curcumin for various therapeutic applications.

## Data Availability

All data included in the article.
